# Disparities in cannabis-related emergency department visits across depressed and non-depressed individuals and the impact of recreational cannabis policy in Ontario, Canada

**DOI:** 10.1017/S0033291723000569

**Published:** 2023-11

**Authors:** Chungah Kim, Gabriel John Dusing, Andrew Nielsen, Frank P. MacMaster, Katherine Rittenbach, Sara Allin, Patricia O'Campo, Tarra L. Penney, Hayley A. Hamilton, Maritt Kirst, Antony Chum

**Affiliations:** 1 Faculty of Health, York University, School of Kinesiology and Health Science, Toronto, Ontario, Canada; 2 Canadian Institute for Health Information, Toronto, Ontario, Canada; 3 University of Calgary, Cumming School of Medicine, Calgary, Alberta, Canada; 4 Faculty of Medicine & Dentistry, Psychiatry Department, University of Alberta, Edmonton, Alberta; 5 University of Toronto, Dalla Lana School of Public Health, Toronto, Ontario; 6 St. Michael's Hospital, Unity Health Toronto, Toronto, Ontario, Canada; 7 Centre for Addiction and Mental Health (CAMH), Toronto, Ontario, Canada; 8 Department of Psychology, Wilfrid Laurier University, Waterloo, Ontario, Canada

**Keywords:** Acute care, Canada, cannabis, cannabis legalisation, depression, emergency department

## Abstract

**Background:**

Recreational cannabis policies are being considered in many jurisdictions internationally. Given that cannabis use is more prevalent among people with depression, legalisation may lead to more adverse events in this population. Cannabis legalisation in Canada included the legalisation of flower and herbs (phase 1) in October 2018, and the deregulation of cannabis edibles one year later (phase 2). This study investigated disparities in cannabis-related emergency department (ED) visits in depressed and non-depressed individuals in each phase.

**Methods:**

Using administrative data, we identified all adults diagnosed with depression 60 months prior to legalisation (*n* = 929 844). A non-depressed comparison group was identified using propensity score matching. We compared the pre–post policy differences in cannabis-related ED-visits in depressed individuals *v.* matched (and unmatched) non-depressed individuals.

**Results:**

In the matched sample (i.e. comparison with non-depressed people similar to the depressed group), people with depression had approximately four times higher risk of cannabis-related ED-visits relative to the non-depressed over the entire period. Phases 1 and 2 were not associated with any changes in the matched depressed and non-depressed groups. In the unmatched sample (i.e. comparison with the non-depressed general population), the disparity between individuals with and without depression is greater. While phase 1 was associated with an immediate increase in ED-visits among the general population, phase 2 was not associated with any changes in the unmatched depressed and non-depressed groups.

**Conclusions:**

Depression is a risk factor for cannabis-related ED-visits. Cannabis legalisation did not further elevate the risk among individuals diagnosed with depression.

## Introduction

Since the legalisation of cannabis in Canada in 2018, many studies have been undertaken to study the effect of legalisation on cannabis-related emergency department (ED) visits and hospitalisations in the general population (Auger et al., 2021; Kim et al., 2022, 2023; Myran et al., 2022*b*; Yeung, Weaver, Janz, Haines-Saah, & Lang, 2020). Given that past-month cannabis use is approximately twice as prevalent among adults with depression compared to those without depression (Gorfinkel, Stohl, & Hasin, 2020), and heavy-use can exacerbate depressive symptoms (Degenhardt, Hall, & Lynskey, 2003), Canadian cannabis legalisation may lead to more adverse events in individuals with depression. This study aims to (1) ascertain the rates of cannabis-related ED-visits in depressed and non-depressed individuals in Ontario, Canada, and (2) fill the gap in the literature regarding the effect of cannabis legalisation on ED-visits in individuals who were diagnosed with depression.

Prior research shows that those with depression in the past year had more severe symptoms of marijuana use disorder based on the Diagnostic and Statistical Manual-5 (compared to those without depression), namely, they used marijuana in larger amounts or over a longer period than intended (OR 1.8, 95% CI 1.56–2.19, *p* < 0.001), repeatedly failed to cut down on the amount of marijuana use (OR 1.9, 95% CI 1.50–2.30, *p* < 0.001), spent an inordinate amount of time on the acquisition, use and recovery of marijuana (OR 1.6, 95% CI 1.41–1.93), and continued to use marijuana despite adverse social consequences (OR 3.2, 95% CI 2.41–4.30) (Dierker, Selya, Lanza, Li, & Rose, 2018). In an analysis of the US National Survey on Drug Use and Health annual surveys (2005–17), a higher prevalence of cannabis use in the past month among people with depression (19%) *v.* those without (9%) was reported (Pacek, Weinberger, Zhu, & Goodwin, 2020). Another study found that depressed mood scores (using the Centre for Epidemiological Studies-Depression scale) were higher for adolescents who reported cannabis use, compared to those who did not (Hernandez et al., 2016). These studies suggest that depression may modify cannabis use such that it elevates its risk of potential adverse events relative to the general population. Increased use of recreational cannabis has been found to be associated with higher risk of cardiovascular adverse events (Singh et al., 2018). There is also evidence that individuals with depression are at higher risk for cannabis-related ED-visits. Using state-wide data from Colorado from 2012 to 14, a study found that individuals with mood disorders (which includes depression and bipolar disorders) have 7.4 times higher prevalence of ED-visits for cannabis use than individuals without (Hall et al., 2018). There is also limited evidence that more liberal cannabis laws may promote cannabis use among depressed individuals: in a study of US adults with mood or anxiety disorders, those living in states with greater access to cannabis through medical licensing laws had 21% higher prevalence of cannabis self-medication compared to those in states without these laws (Sarvet et al., 2018).

**Figure 1.**
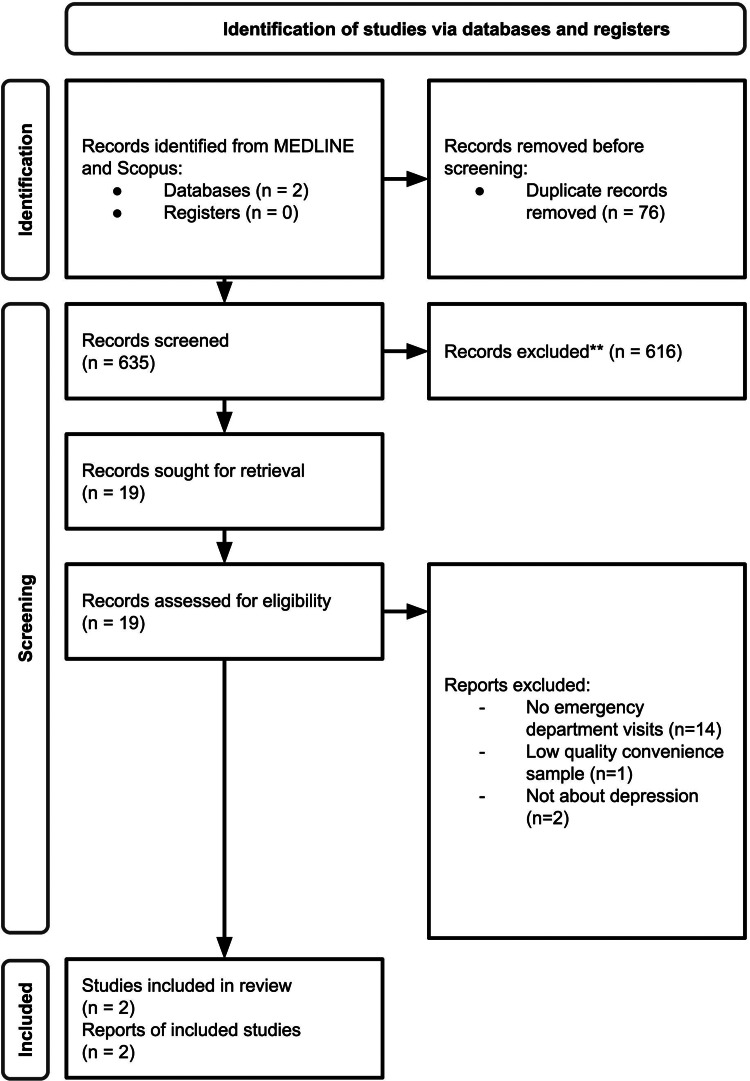
PRISMA flowchart and search terms. Scopus TITLE-ABS-KEY (“depress*” OR “dysthymia” OR “mood disorder”) AND TITLE-ABS-KEY (“cannabis” OR “marijuana”) AND ALL (“emergency service” OR “emergency department” OR “emergency room”) Pubmed. ((depress*[Title/Abstract]) OR (dysthymia[Title/Abstract]) OR (mood?disorder[Title/Abstract])) AND ((cannabis[Title/Abstract]) OR (marijuana[Title/Abstract])) AND ((emergency?service) OR (emergency?department) OR (emergency?room))

To establish the unique contribution of our study to the literature, a comprehensive literature search using MEDLINE and Scopus was conducted (1) to identify prior studies that quantified cannabis-related ED-visits in individuals with depression, and (2) the impact of cannabis legalisation on cannabis-related ED-visits among those with depression. Cannabis-related ED visits is defined as any ED visit for mental and behavioural disorder and/or poisoning due to cannabis use. See Fig. 1 for PRISMA flowchart and search terms. Only two studies met our search criteria. In a Colorado study of a single hospital system that included 4202 cannabis-related ED and urgent care visits among adolescents, a comorbid psychiatric diagnosis was made in 71% of the sample, with the highest diagnoses for depression (39%), mood disorder (22%), anxiety disorder (13%) and bipolar disorder (6%) (Wang, Davies, Halmo, Sass, & Mistry, 2018). This study provides evidence that depression is likely the most common psychiatric condition among adolescents with cannabis-related ED-visits. A single-centre study involving retrospective chart review of cannabis-related ED-visits found that patients with depression accounted for 4.3% of visits 2020 (Shelton et al., 2020). Both studies were limited by (1) convenience sampling, (2) samples drawn from a single-centre/hospital and (3) the lack of population-wide state-level data, which may increase the risk of selection bias and reduce generalisability. Furthermore, no studies investigated the potential differential impact of cannabis legalisation on depressed *v.* non-depressed individuals.

Based on the results of our systematic search, this is the first study to characterise the risk of cannabis-related ED-visits in depressed and non-depressed populations using population-wide data, and is also the first study to compare the impact of cannabis legalisation on the cannabis-related ED-visits in depressed and non-depressed individuals. In Ontario, legalisation was multi-phased, with the legalisation of flower and herbs (phase 1) in October 2018, and the legalisation of cannabis edibles one year later (phase 2). Given the prior literature which indicates the increased vulnerability of depressed individuals regarding cannabis use, we hypothesise that (1) cannabis-related ED-visits are greater in depressed *v.* non-depressed individuals, and (2) the effects of recreational cannabis legalisation on cannabis-related ED-visits is greater among those with depression. Since prior studies have shown gender differences in the rates and severity of cannabis use disorders (Dierker et al., 2018), we tested these hypotheses for men and women separately accounting for potential differences in the effects of legalisation across men and women.

## Methods

We performed a comparative interrupted time-series (CITS) analysis to examine whether the effect of Ontario's cannabis legalisation on cannabis-related ED-visits differed between depressed and non-depressed individuals.

### Data source and study population

Using health administrative data accessed through ICES (formerly the Institute for Clinical Evaluative Sciences), we created a cohort that included the entire adult population (18+) eligible for Ontario Health Insurance Plan, OHIP (*n* = 11 156 100) on 17 October 2015, and they were followed through their healthcare records until 20 May 2021. OHIP is Ontario's universal healthcare that covers over 95% of Ontario residents, those who are not eligible includes individuals in their 3-month waiting periods and migrants with temporary status (e.g. international students, temporary workers) (Ontario Ministry of Health, 2022), which includes approximately 500 000 people (OHIP For All, 2022). Participants must be 18 or over as of 17 October 2018 (the date of cannabis legalisation), and have continuous OHIP coverage and residency in Ontario for the entire study period to be included in the study (October 2015 to May 2021).

This study complied with privacy regulations of ICES. To protect privacy, all cell sizes (i.e. the number of individuals in a categorical variable) with fewer than six individuals in all descriptive tables were suppressed and reported as *n* < 6. Consent was not obtained for participants for the use of their data in this study. ICES is an independent, non-profit research institute whose legal status under Ontario's health information privacy law allows it to collect and analyse healthcare and demographic data used for the study, without consent, for the purposes of health system evaluation and improvement. All patient information was anonymised and de-identified prior to analysis. Ethics approval for this study was obtained through York University (REB# 2022-254).

### Population at risk

We identified individuals diagnosed with depression by a physician [OHIP diagnostic code 311 (Government of Ontario, 2015)] in a look back period up to 5 years prior to legalisation between 17 October 2013 to 16 October 2018 (*n* = 929 844). This look back period was established based on consultations with two clinical psychiatrists, given that depression diagnoses that are older than 5 years are likely to indicate an illness that has been resolved, while diagnoses within 5 years may indicate unresolved illness. A prior study has shown that the code has high specificity and positive predictive value for the measure of depressive disorder (i.e. 99.69% specificity and 89.62% for positive predictive value) (Alaghehbandan, MacDonald, Barrett, Collins, & Chen, 2012).

### Exposure: cannabis-related policy in Ontario

To examine the impact of recreational cannabis policy on cannabis-related ED-visits among individuals diagnosed with depression (hypothesis #2), we used three distinct time periods related to the Ontario legal cannabis availability: (1) the pre-legalisation period (October 2015–October 2018), (2) phase 1 of legalisation (October 2018–February 2020), when flower and herb were available online through a government website and at limited private physical retail locations, and (3) phase 2 (March 2020 onwards), which marked the rapid private retail expansion (removal of provincial retailer cap) alongside increased edible cannabis availability. These time periods have been used in prior studies on cannabis-related ED-visits following cannabis legalisation and commercialisation in the Ontario population (Kim et al., 2022). Cannabis-related ED-visits between March and April 2020 were censored to account for significant decreases in healthcare utilisation at the onset of the COVID-19 pandemic, as suggested by prior literature (Myran et al., 2022*a*).

### Outcome

The outcome of the study was monthly cannabis-related ED-visits from October 2015 to May 2021 in Ontario (the frequency of the event by gender for each month of the study period). We define cannabis-related ED visits as any ED visit for mental and behavioural disorders and poisonings due to cannabis use. Monthly cannabis-related ED-visits were collected from the National Ambulatory Care Reporting System (NACRS) using at least one of the following ICD-10 codes for either the primary or supplemental diagnosis: F12 (including 11 subcodes for mental and behavioural disorders related to cannabis) and T40.7 (cannabis poisoning) (Canadian Institute for Health Information, 2021*a*). Positive cases were identified using the primary and non-primary diagnosis codes in NACRS. This method of extracting cannabis-related acute care events conforms with the definition set out for the Canadian Institute for Health Information (Canadian Institute for Health Information, 2021*b*), which has been used in prior studies (Callaghan et al., 2021; Counsil of State and Territorial Epidemiologists, n.d.; Myran et al., 2022*b*; Yeung et al., 2020; Yeung, Weaver, Hartmann, Haines-Saah, & Lang, 2021), and shows a high positive predictive value (>95%) (DeYoung et al., 2017).

### Statistical analysis

To compare the depressed population with non-depressed individuals (hypothesis #1), we conducted a matching method to identify an appropriate counterfactual, i.e. a sample of the population that has not been diagnosed with depression that is matched on key variables to individuals diagnosed with depression. We used 1-to-1 nearest-neighbour propensity score matching on (1) age group, (2) sex, (3) neighbourhood socioeconomic status [using the Ontario Marginalisation Index (Public Health Ontario, 2022)], (4) residency in Northern or Southern Ontario and (5) seven Aggregated Diagnosis Groups (ADGs) clusters, produced by the Johns-Hopkins Adjusted Clinical Group® System (version 10), which are used as our comorbidity indicators (John Hopkins University, 2022). Northern *v.* southern indicator was included because prior research has found that individuals living in Northern Ontario have higher rates of cannabis-related ED-visits (Kim et al., 2022).

After matching, we produced age- and sex-specific rates for cannabis-related ED-visits separated for the depressed and non-depressed groups in matched and unmatched samples. For comparison of the outcome between depressed and non-depressed individuals, the exact Poisson method was used to calculate incidence rate ratios (IRR) with 95% confidence intervals (CI), which presents effect sizes. These comparisons were performed separately for men and women, and on the matched and unmatched samples.

To investigate the potential differential impact of legalisation across the depressed and non-depressed groups (hypothesis #2), we conducted a CITS analysis for cannabis-related ED-visits between depressed and non-depressed individuals using the user-written package for ITSA (Linden, 2015):
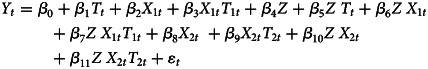
where *Y_t_* is the monthly count of cannabis-related ED events at time-point *t*. *Z* is the dummy variable denoting cohort assignment status [depressed (=1) *v.* not depressed (=0)], while *X* is the dummy variable denoting cannabis legalisation phases, with *X*_1*t*_ for phase 1 of legalisation and *X*_2*t*_ for phase 2. *T_t_* is the time since the start of the study at time point *t* and *X_t_T_t_* is an interaction term. Detailed descriptions of the dummy variables *X*_1*t*_ and *X*_12_, and continuous variables *T_t_*, *T*_1*t*_ and *T*_2*T*_ are presented in online Supplementary Tables S2 and S3. Betas 1, 2, 3, 8, 9 refer to parameters for the control group (i.e. non-depressed), and betas 5, 6, 7, 10, 11 are used to modify the former coefficients to estimate the parameters of interest for the depressed group. For example, change in slope in the pre-legalisation period for the non-depressed group is represented by *β*_1_, while *β*_1_ and *β*_5_ are summed to represent pre-legalisation slope for the depressed group. *β*_4_ in the above represents the difference in the level (intercept) of the outcome variable between target and comparison prior to the intervention; however, it is not used in the parameter estimation because it is not of interest in our study. See details in the online Supplementary Table S1 for the full explanations for every parameter that are used in our analyses. Four gender-stratified models were specified, where model 1 is the males-only model using the unmatched sample, model 2 is the female unmatched model, model 3 is the matched male model and model 4 is the matched female model. STATA16 (College Station, TX) was used for our data analysis. Each test (per every postestimation analysis) was two-tailed with a 5% as alpha to test statistical significance. To correct for multiple testing, we estimated the probability of making at least one type 1 error to be 53%. The overall level of significance was calculated as 0.0033 using the Bonferroni correction. We present both the matched and unmatched results since matched results compare depressed with a non-depressed group similar to the depressed group, while the unmatched results compare the depressed with the non-depressed general population. While the matched results highlight the effects of independent effects of depression, the latter highlights average (unadjusted) differences that are relevant for healthcare planning.

### Sensitivity analyses

There may be a difference in healthcare utilisation patterns between individuals whose ED-visit lists cannabis as either the primary diagnosis, or as one of the secondary diagnoses. To ensure the consistency of our results, we repeated our analysis where the outcome is restricted to ED-visits with cannabis listed as the primary diagnosis (i.e. removing cases with cannabis as secondary diagnosis only).

## Results

The most common ICD-10 code used for cannabis-related ED-visits was F12.1 (mental/behavioural disorder from harmful use), which was found in 52% of those with depression and 48% of those without depression. The second and third most common codes, F12.0 (mental/behavioural disorder from acute intoxication) and T40.7 (poisoning) respectively, were more prevalent among those without depression than those with depression (see Table 1 for code distribution in the matched and unmatched samples) (Table 1).

**Table 1.** Percent of total cannabis-related ED-visits by ICD-10-CA codes, depressed *v.* non-depressed, in matched and unmatched sample

ICD-10-CA	Definition	Matched sample: individuals with depression	Matched sample: individuals without depression	Unmatched sample: individuals with depression	Unmatched sample: individuals without depression
**F12**	Mental and behavioural disorders associated with cannabis				
F12.0	Mental and behavioural disorders due to use of cannabinoids: acute intoxication	14.11%	18.31%	13.88%	21.12%
F12.1	Mental and behavioural disorders due to use of cannabinoids: harmful use	51.97%	47.56%	51.79%	49.02%
F12.2	Mental and behavioural disorders due to use of cannabinoids: dependence syndrome	9.61%	5.97%	10.93%	5.64%
F12.3	Mental and behavioural disorders due to use of cannabinoids: withdrawal state	1.50%	1.29%	1.49%	1.40%
F12.4	Mental and behavioural disorders due to use of cannabinoids: withdrawal state with delirium	0.02%	0.02%	0.02%	0.02%
F12.5	Mental and behavioural disorders due to use of cannabinoids: psychotic disorder	7.61%	7.13%	7.69%	4.91%
F12.6	Mental and behavioural disorders due to use of cannabinoids: amnesic syndrome	0.14%	0.13%	0.14%	0.06%
F12.7	Mental and behavioural disorders due to use of cannabinoids: residual and late-onset psychotic disorder	0.09%	0.08%	0.10%	0.05%
F12.8	Mental and behavioural disorders due to use of cannabinoids: other mental and behavioural disorders	1.81%	2.13%	1.88%	1.49%
F12.9	Mental and behavioural disorders due to use of cannabinoids: unspecified mental and behavioural disorder	5.99%	7.47%	6.17%	4.35%
**T40.7**	Poisoning by, adverse effect of cannabis (derivatives)	9.69%	12.59%	9.84%	13.09%

Note: percentage add up to more than 100% because some hospitalisation records had more than one cannabis-related diagnostic code. 2.07% of hospitalisations included 2+ cannabis-related diagnoses.

Table 2 presents the sample characteristics for unmatched and matched samples at baseline (Table 2). Association between each exposure and depression status is obtained through logistic regression. The strength and direction of the association is summarised by Somer's *D*, where a higher absolute value represents a stronger association. It should be noted that the matching procedure reduced the association between every covariate and depression status (as indicated by a lower Somer's *D*), which implies an improved covariate balance between the target and comparison groups in the matched sample. For example, in the unmatched sample, the proportion of women in the target (depressed) and comparison (non-depressed) group was 62% and 50% respectively, as opposed to 62% and 62% in the matched sample. For further information on the differences between the matched and unmatched groups in terms of the proportion of those with cannabis-related ED visits across depression and substance use disorder statuses, please see the online Supplementary Tables S4 and S5.

**Table 2.** Descriptive characteristics of unmatched and matched study samples at baseline

	Unmatched			Matched		
	Comparison (*n* = 11 163 039)	Target (*n* = 929 844)	Somer's D, *p* value^a^	Comparison (*n* = 916 146)	Target (*n* = 916 146)	Somer's D, *p* value^a^
Age group			0.066, *p* < 0.0001			0.004, *p* < 0.0001
18–24	1 139 095 (10.2%)	121 976 (13.1%)		116 062 (12.7%)	118 092 (12.9%)	
25–64	7 645 791 (68.5%)	652 121 (70.1%)		642 920 (70.2%)	643 633 (70.3%)	
65+	2 408 153 (21.6%)	155 747 (16.7%)		157 164 (17.2%)	154 421 (16.9%)	
Gender (raw, %)			0.124, *p* < 0.0001			0.001, *p* = 0.4494
Men	5 575 006 (49.9%)	348 975 (37.5%)		344 757 (37.6%)	344 261 (37.6%)	
Women	5 588 033 (50.1%)	580 869 (62.5%)		571 389 (62.4%)	571 885 (62.4%)	
Deprivation quintile (raw, %)			0.053, *p* < 0.0001			0.012, *p* < 0.0001
1	2 519 566 (22.6%)	191 345 (20.6%)		172 102 (18.8%)	159 940 (17.5%)	
2	2 315 422 (20.7%)	179 130 (19.3%)		156 404 (17.1%)	154 862 (16.9%)	
3	2 125 738 (19.0%)	168 599 (18.1%)		160 525 (17.5%)	162 217 (17.7%)	
4	2 058 596 (18.4%)	176 258 (19.0%)		174 843 (19.1%)	180 586 (19.7%)	
5	2 071 153 (18.6%)	208 396 (22.4%)		252 272 (27.5%)	258 541 (28.2%)	
Missing	72 564 (0.7%)	6116 (0.7%)		0 (0.0%)	0 (0.0%)	
Instability (raw, %)			0.077, *p* < 0.0001			0.019, *p* < 0.0001
Q1	2 376 024 (21.3%)	160 789 (17.3%)		185 484 (20.2%)	190 289 (20.8%)	
Q2	2 072 860 (18.6%)	155 882 (16.8%)		175 310 (19.1%)	177 881 (19.4%)	
Q3	1 990 547 (17.8%)	163 461 (17.6%)		170 618 (18.6%)	167 397 (18.3%)	
Q4	1 980 547 (17.7%)	182 305 (19.6%)		180 420 (19.7%)	174 611 (19.1%)	
Q5	2 668 286 (23.9%)	261 291 (28.1%)		204 314 (22.3%)	205 968 (22.5%)	
Missing	72 564 (0.7%)	6116 (0.7%)		0 (0.0%)	0 (0.0%)	
Dependency			0.023, *p* < 0.0001			0.014, *p* < 0.0001
Q1	2 939 450 (26.3%)	230 811 (24.8%)		225 260 (24.6%)	229 197 (25.0%)	
Q2	2 261 385 (20.3%)	186 685 (20.1%)		179 574 (19.6%)	185 172 (20.2%)	
Q3	1 962 249 (17.6%)	163 699 (17.6%)		160 733 (17.5%)	162 293 (17.7%)	
Q4	1 889 207 (16.9%)	159 123 (17.1%)		161 216 (17.6%)	157 679 (17.2%)	
Q5	2 038 184 (18.3%)	186 410 (20.0%)		189 363 (20.7%)	181 805 (19.8%)	
Missing	72 564 (0.7%)	6116 (0.7%)		0 (0.0%)	0 (0.0%)	
ADG3 (time limited: major)			0.009, *p* < 0.0001			0.001, *p* < 0.0001
Yes	919 814 (8.2%)	16 673 (1.8%)		10 279 (1.1%)	9424 (1.0%)	
No	11 146 366 (99.9%)	10 030 (1.1%)		905 867 (98.9%)	906 722 (99.0%)	
ADG4 (time limited: major-primary infections)			0.005, *p* < 0.0001			<0.001, *p* = 0.0066
Yes	8116 (0.1%)	5195 (0.6%)		5072 (0.6%)	4803 (0.5%)	
No	11 154 923 (99.9%)	924 649 (99.4%)		911 074 (99.4%)	911 343 (99.5%)	
ADG9 (likely to recur: progressive)			0.005, *p* < 0.0001			0.001, *p* < 0.0001
Yes	13 698 (0.1%)	5636 (0.6%)		5906 (0.6%)	5385 (0.6%)	
No	11 149 341 (99.9%)	924 208 (99.4%)		910 240 (99.4%)	910 761 (99.4%)	
ADG11 (chronic medical: unstable)			0.015, *p* < 0.0001			0.003, *p* < 0.0001
Yes	40 469 (0.4%)	17 528 (1.9%)		19 277 (2.1%)	16 978 (1.9%)	
No	11 122 570 (99.6%)	912 316 (98.1%)		896 869 (97.9%)	899 168 (98.1%)	
ADG16 (chronic specialty: unstable-orthopaedic)			0.001, *p* < 0.0001			<0.001, *p* = 0.4930
Yes	1486 (0.0%)	708 (0.1%)		653 (0.1%)	678 (0.1%)	
No	11 161 553 (100.0%)	929 136 (99.9%)		915 493 (99.9%)	915 468 (99.9%)	
ADG22 (injuries/adverse effects: major)			0.028, *p* < 0.0001			0.001, *p* = 0.0011
Yes	20 477 (0.2%)	27 847 (3.0%)		19 456 (2.1%)	20 100 (2.2%)	
No	11 142 862 (99.8%)	901 997 (97.0%)		896 690 (97.9%)	896 046 (97.8%)	
ADG32 (malignancy)			0.002, *p* < 0.0001			0.001, *p* < 0.0001
Yes	6536 (0.1%)	2472 (0.3%)		3037 (0.3%)	2351 (0.3%)	
No	11 156 503 (99.9%)	927 372 (99.7%)		913 109 (99.7%)	913 795 (99.7%)	

a Association between each target and comparison status (i.e. depressed *v*. non-depressed) is obtained through logistic regression. The strength and direction of logistic regression is summarised by Somer's *D*, where a higher absolute value represents a stronger association.

Rates of cannabis-related ED-visits by gender and depression status in each phase of cannabis-related policy are presented in Table 3 (matched and unmatched). Depressed individuals have consistently higher rates of cannabis-related ED-visits for both matched and unmatched samples, but the difference in the rates between depressed and non-depressed individuals is greater in the unmatched sample (7–8 times higher), compared to the matched sample (4 times greater) as indicated by the IRR value found in Table 3.

**Table 3.** Rates of cannabis-related ED-visits per 100 000 person-years in depressed and non-depressed individuals (both matched and unmatched sample)

	Entire study period (18 Oct 2015–17 Jul 2021)	Pre-legalisation period (17 Oct 2015–17 Oct 2018)	Phase 1 (18 Oct 2018–17 Mar 2020)	Phase 2 (18 Mar 2020–17 May 2021)
Matched sample				
Depressed men (344 261)	49.932	49.617	50.038	51.088
Non-depressed men (344 757)	11.359	10.818	11.880	12.364
Chi-square – test of proportion	IRR: 4.417 95 CI 3.105–6.424 *p* < 0.0001	IRR: 4.628 95 CI 3.228–6.795 *p* < 0.0001	IRR: 4.201 95 CI 2.974–6.060 *p* < 0.0001	IRR: 4.029 95 CI 2.871–5.766 *p* < 0.0001
Depressed women (571 885)	20.760	18.545	23.173	24.065
Non-depressed women (571 389)	5.060	3.827	6.141	7.657
Chi-square – test of proportion	IRR: 4.100 95 CI 2.713–6.383 *p* < 0.0001	IRR: 4.814 95 CI 3.020–8.005 *p* < 0.0001	IRR: 3.797 95 CI 2.600–5.679 *p* < 0.0001	IRR: 3.134 95 CI 2.217–4.506 *p* < 0.0001
Unmatched sample				
Depressed men (348 975)	52.178	52.556	51.480	52.439
Non-depressed men (5 575 006)	6.133	4.556	7.791	8.661
Chi-square – test of proportion	IRR: 8.502 95 CI 7.063–10.206 *p* < 0.0001	IRR: 11.573 95 CI 9.520–14.046 *p* < 0.0001	IRR: 6.626 95 CI 5.538–7.901 *p* < 0.0001	IRR: 6.053 95 CI 5.078–7.190 *p* < 0.0001
Depressed women (580 869)	22.335	20.310	24.663	25.006
Non-depressed women (5 588 033)	3.310	2.244	4.370	5.192
Chi-square – test of proportion	IRR: 6.760 95 CI 5.360–8.506 *p* < 0.0001	IRR: 9.009 95 CI 6.949–11.674 *p* < 0.0001	IRR: 5.638 95 CI 4.554–6.959 *p* < 0.0001	IRR: 4.810 95 CI 3.913–5.891 *p* < 0.0001

IRR, incidence rate ratio.

While the initial disparities between depressed and non-depressed individuals are large, they appear to reduce over the course of the study period. In the matched sample in Table 3, depressed women were 4.81 (95% CI 3.02–8.00) times more likely to have cannabis-related ED-visits compared to non-depressed women during the pre-legalisation period. This rate fell to 3.80 during phase 1, and fell yet again in phase 2 to 3.13 (95% CI 2.22–4.51). This pattern of falling IRRs is repeated among men, with depressed men having cannabis-related ED-visits at a rate 4.63 times higher than non-depressed men during the pre-legalisation period, 4.20 during phase 1 and 4.03 in phase 2. A similar pattern of decreasing disparities is also seen in the unmatched sample (Table 3).

With regards to the CITS analyses, in the matched sample (Table 4), trends in ED-visits among depressed individuals are compared to those who were as similar as possible to them without depression. While there was modest month-to-month increase in the number of ED-visits in the pre-legalisation period in both depressed and non-depressed men and women (ranging from 1% to 2% per month), neither legalisation nor the introduction of edibles led to further sudden or gradual changes (as shown in Table 4). There was also no difference in the size or direction of change observed across the target and comparison groups.

**Table 4.** Comparative interrupted time-series analyses, estimations based on a multiple group, multiple intervention design for both matched and unmatched samples

	Depressed (A)	Non-depressed (B)	Difference test (A)–(B)
Male, matched sample			
Pre-legalisation trend	0.016*** (0.011 to 0.020)	0.017** (0.007 to 0.026)	−0.001 (−0.012 to 0.009)
Phase 1 intercept change	−0.220 (−0.478 to 0.079)	−0.021 (−0.043 to 0.002)	−0.199 (−0.478 to 0.079)
Phase 1 slope change	−0.008 (−0.019 to 0.002)	−0.004 (−0.024 to 0.017)	−0.005 (−0.028 to 0.018)
Phase 2 intercept change	−0.076 (−0.644 to 0.492)	0.018 (−0.018 to 0.055)	−0.094 (−0.653 to 0.465)
Phase 2 slope change	0.013 (−0.024 to 0.049)	0.015 (−0.017 to 0.046)	−0.002 (−0.050 to 0.046)
Female, matched sample			
Pre-legalisation trend	0.015*** (0.011 to 0.018)	0.017*** (0.011 to 0.023)	−0.002 (−0.009 to 0.005)
Phase 1 intercept change	0.043 (−0.241 to 0.328)	−0.011 (−0.028 to 0.007)	0.054 (−0.222 to 0.330)
Phase 1 slope change	−0.007 (−0.020 to 0.006)	0.006 (−0.010 to 0.023)	−0.013 (−0.034 to 0.008)
Phase 2 intercept change	0.132 (−0.318 to 0.581)	0.004 (−0.028 to 0.037)	0.127 (−0.307 to 0.562)
Phase 2 slope change	0.008 (−0.018 to 0.034)	0.010 (−0.019 to 0.040)	−0.003 (−0.042 to 0.037)
Male, unmatched sample			
Pre-legalisation trend	0.015*** (0.012 to 0.018)	0.020*** (0.017 to 0.023)	−0.005* (−0.010 to −0.001)
Phase 1 intercept change	−0.206** (−0.358 to −0.054)	0.231*** (0.131 to 0.331)	−0.437*** (−0.619 to −0.255)
Phase 1 slope change	−0.008 (−0.022 to 0.007)	−0.006 (−0.014 to 0.002)	−0.002 (−0.018 to 0.014)
Phase 2 intercept change	0.044 (−0.217 to 0.305)	0.089 (−0.193 to 0.370)	−0.044 (−0.429 to 0.340)
Phase 2 slope change	0.006 (−0.017 to 0.030)	0.011 (−0.016 to 0.038)	−0.005 (−0.041 to 0.030)
Female, unmatched sample			
Pre-legalisation trend	0.016*** (0.011 to 0.019)	0.023*** (0.019 to 0.027)	−0.007* (−0.013 to −0.002)
Phase 1 intercept change	−0.036 (−0.170 to 0.097)	0.261** (0.111 to 0.411)	−0.297** (−0.498 to −0.097)
Phase 1 slope change	−0.007 (−0.018 to 0.003)	−0.003 (−0.014 to 0.007)	−0.004 (−0.019 to 0.011)
Phase 2 intercept change	0.021 (−0.421 to 0.463)	0.176 (−0.154 to 0.507)	−0.155 (−0.707 to 0.396)
Phase 2 slope change	0.013 (−0.027 to 0.053)	0.011 (−0.019 to 0.041)	0.002 (−0.048 to 0.052)

In the unmatched sample, trends in ED-visits among depressed individuals are compared to the Ontario general population (see the online Supplementary Table S1 for the complete description of the following coefficients). A similar upward pre-legalisation trend was observed in the target (*β*_1_ + *β*_5_) and comparison (*β*_1_) group; however, in both men and women, the upward trajectory (*β*_5_) was stronger in the non-depressed group, i.e. an additional 0.5% increase was seen in non-depressed men, and an additional 0.7% in non-depressed women, relative to their depressed counterparts. The immediate change (*β*_4_) associated with phase 1 (Table 4) appeared to have divergent impact in the target and comparison groups. For men, there was an immediate 20.6% reduction (95% CI 5.4–35.8% reduction) in the depressed group, while non-depressed men saw a 23.1% increase. For women, phase 1 was not associated with any change in ED-visits in the depressed group, while the non-depressed group saw a 26.1% immediate increase (95% CI 11.1–41.1%). The difference tests (*β*_4_) in the immediate change found that there were statistically significant differences between non-depressed and depressed individuals for both men and women.

The results of the supplementary analysis where the outcome was ED-visits with cannabis listed as the primary outcome in online Supplementary Table S6. The results show that removing cases where cannabis was secondary diagnosis only, the sensitivity test showed substantially similar findings compared to our main analyses.

## Discussion

We found that individuals diagnosed with depression have consistently higher rates of cannabis-related ED-visits compared to those without for both matched and unmatched samples: approximately four times higher risk based on the matched sample (11 *v.* 50 events/100 000 person-years in men, 5 *v.* 20 events/100 000 person-years in women), which provides support for hypothesis #1. Phase 1 and 2 legalisation was not associated with significant changes in ED-visits in the matched sample; however, immediate increase was associated with phase 1 in the unmatched sample in the non-depressed group (i.e. the general population). This immediate increase (23% in men, 26% in women) in the general population is consistent with prior literature on the subject. For example, Myran et al., also based in Ontario, found an immediate increase of 12% in rates of cannabis-attributable ED-visits in the general population (Myran et al., 2022*b*). The difference across the studies may be attributable to different age cut-offs used, and the fact that individuals diagnosed with depression were not included in our comparison group.

On the other hand, contrary to our hypothesis #2, depressed individuals did not experience any increase in ED-visits associated with legalisation. This is the first study to examine cannabis-related acute care rates and disparities in depressed *v.* non-depressed individuals. Despite the prior evidence that depressed individuals are more vulnerable to cannabis-related disorders and problematic use, our study shows that this group did not experience increased risk of cannabis-related ED-visits due to the recreational cannabis legalisation. Prior research shows that a proportion of people with depression may be using cannabis for self-medication purposes (i.e. to help cope with symptoms of depression) (Wallis et al., 2022), and many are regular users of cannabis even prior to legalisation, and thus the legalisation for recreational use may not have a significant effect on their cannabis-related ED outcome. Further research should investigate whether self-medicating with cannabis among depressed individuals is a major risk factor for cannabis-related ED visits.

The discrepancy between the matched and the unmatched sample may be interpreted as adjusted *v.* unadjusted results, this is because the matching procedure balances the distribution of sociodemographic and health characteristics across the target and comparison groups, and subsequently we can estimate, *ceteris paribus*, the independent effects of legalisation by depression status. On the other hand, comparison to the general population (shown in our unmatched sample results), which gives the average risk of cannabis-related ED-visits in the depressed *v.* the average risk in the general population, can also be informative since it can better represent the disparities between the two groups regardless of their sociodemographic and health information.

The limitations of this study are as follows: (1) the depressed group that we identified would not include undiagnosed patients with depression, who may be hidden in the general population. Since they have not been clinically assessed, their symptoms may be worse or better than those diagnosed, which may bias the results towards an unknown direction. (2) Although our use of CITS may have reduced the effects of unobservable concurrent interventions, there is still possibility that the unobservable confounders have differential effects for the target and comparison groups. (3) Although we had the censoring window between March and April 2020 to account for the effects of the COVID-19 pandemic on healthcare utilisation, this could bias the results towards an unknown direction if there were differential effects between depressed and non-depressed individuals. (4) There is a possibility that our matching method may introduce dependency among the data which is a known issue with propensity score-based matching methods. However, given that unmatched and matched samples produced similar results, our use of matching is likely robust.

## Conclusion

Individuals diagnosed with depression have a higher rate of cannabis-related ED-visits; however, legalisation did not appear to be associated with further elevated risk in ED-visits for such individuals. The general elevated risk of cannabis misuse in this group is still a concern, and healthcare providers may consider screening for cannabis use in individuals diagnosed with depression, and provide adequate resources and education to reduce the risk of cannabis-related acute care. Furthermore, while the legalisation of herbs and flowers (phase 1) and edibles (phase 2) was not associated with greater increases of cannabis-related acute care in the depressed *v.* general population, there is a need to continue monitoring this clinical group given the expansion of the cannabis market in Canada, which have led to the introduction of new edible products such as cannabis-infused coffee, beers, spirits, chocolates and desserts and new methods of delivery (e.g. cannabis patches, vapes, suppositories) that are now being marketed with greater choice than ever before. In addition, future studies may take into account the impacts of the market regulation for cannabis on depressed and non-depressed individuals. For example, while this study only included Ontario, where the online and wholesale distribution of cannabis products is centralised through a crown corporation monopoly (i.e. Ontario Cannabis Store), future studies could compare with other jurisdictions that have a decentralised approach (e.g. Alberta).

## Supporting information

Kim et al. supplementary material Kim et al. supplementary material

## Data Availability

The data used in this study are third-party data that were provided by ICES (formerly known as the Institute for Clinical Evaluative Sciences), and were accessed remotely through their secure Data & Analytics Services. While data sharing agreements prohibit ICES from making the dataset publicly available, access may be granted to those who meet pre-specified criteria for confidential access, please contact a representative at das@ices.on.ca to request access and verification. The full dataset creation plan and underlying analytics code are available from the authors upon request, understanding that the computer programmes may rely upon coding templates or macros that are unique to ICES and are therefore either inaccessible or may require modification.

## Supplementary material

The supplementary material for this article can be found at https://doi.org/10.1017/S0033291723000569.

## Permission to reproduce material from other sources

Not applicable as no materials were reproduced from other sources.
