# Supplementary material for: Disparities in cannabis-related emergency department visits across depressed and non-depressed individuals and the impact of recreational cannabis policy in Ontario, Canada
Source: Psychol Med. 2023 Jun 22;53(15):7127–37. doi: 10.1017/S0033291723000569 (PMC10719623; doi:10.1017/S0033291723000569)
Supplement: Kim et al. supplementary material [file S0033291723000569sup001.docx]

**Supplementary table 1: Regression coefficients parameters of interest**

| Model used for comparative interrupted time series with 2 groups and 2 interruptions:  $Y_{t} = \beta_{0}+\beta_{1}T_{t}+\beta_{2}X_{1t}+\beta_{3}X_{1t}T_{1t}+\beta_{4}Z+\beta_{5}ZT_{t}+\beta_{6}ZX_{1t}+\beta_{7}ZX_{1t}T_{1t}+\beta_{8}X_{2t}$  $+\beta_{9}X_{2t}T_{2t}+\beta_{10}ZX_{2t}+\beta_{11}ZX_{2t}T_{2t}+\varepsilon_{t}$ | |
| --- | --- |
| $\beta_{0}$ | Grand intercept |
| $\beta_{1}$ | Pre-legalization slope (non-depressed) |
| $\beta_{1}$+ $\beta_{5}$ | Pre-legalization slope (depressed) |
| $\beta_{2}$ | Phase 1 intercept change (non-depressed) |
| $\beta_{2}$+ $\beta_{6}$ | Phase 1 intercept change (depressed) |
| $\beta_{1}$+ $\beta_{3}$ | Phase 1 slope (non-depressed) |
| $\beta_{1}$+ $\beta_{3}$+$\beta_{5}$+ $\beta_{7}$ | Phase 1 slope (depressed) |
| $\beta_{8}$ | Phase 2 intercept change (non-depressed) |
| $\beta_{8}$+$\beta_{10}$ | Phase 2 intercept change (depressed) |
| $\beta_{1}$+ $\beta_{3}$+$\beta_{9}$ | Phase 2 slope (non-depressed) |
| $\beta_{1}$+ $\beta_{3}$+$\beta_{5}$+ $\beta_{7}$+$\beta_{9}$+ $\beta_{11}$ | Phase 2 slope (depressed) |
| ɛ_t_ | Error |

**Supplementary Table 2: Description of the indicator variables X_1t_ and X_2t_**

|  | Pre-legalisation period | Phase 1 of legalisation | Phase 2 of legalisation |
| --- | --- | --- | --- |
| X_1t_ | 0 | 1 | 0 |
| X_2t_ | 0 | 1 | 1 |

**Supplementary Table 3: Description of the continuous variables T_t_, T_1t_, and T_2T_**

| T_t_ | Time since the start of the pre-legalisation period |
| --- | --- |
| T_1t_ | Time since the start of Phase 1 of legalisation |
| T_2T_ | Time since the start of Phase 2 of legalisation |

**Supplementary Table 4: Proportion of individuals with substance use disorder among depressed and nondepressed individuals: before and after propensity score matching**

| Unmatched Sample | | | |
| --- | --- | --- | --- |
|  | | Substance Use Disorder Diagnosis | |
|  |  | No | Yes |
| Depression Diagnosis | No (n=11,163,039) | 98.34 | 1.66 |
|  | Yes (n=929,844) | 91.75 | 8.25 |
| Matched Sample | | | |
|  | | Substance Use Disorder Diagnosis | |
|  |  | No | Yes |
| Depression Diagnosis | No (n=916,146) | 97.88 | 2.12 |
|  | Yes (n=916,146) | 92.04 | 7.96 |

Interpretation: the relative stability of these proportions (within each depression status group) before and after the matching provides some evidence that the matching process did not bias our results by selecting those with higher/lower risk within each depression status group.

**Supplementary Table 5: Proportion of individuals with a cannabis related ED visit across depression and substance use disorder statuses, using data from the matched sample**

|  | Cannabis related ED visit (row percent) | |
| --- | --- | --- |
|  | No | Yes |
| Nondepressed; no substance use disorder diagnosis (n=896,690) | 99.62% | 0.38% |
| Depressed; no substance use disorder diagnosis (n=843,204) | 98.69% | 1.31% |
| Nondepressed; substance use disorder diagnosis (n=19,456) | 92.95% | 7.05% |
| Depressed; substance use disorder diagnosis (n=72,942) | 87.37% | 12.63% |

Interpretation: Both depression and substance use disorder appear to contribute to the risk of a cannabis-related ED visit. Those with both depression and substance use disorder are at the highest risk.

**Supplementary Table 6: Comparative interrupted time-series analyses, estimations based on a multiple group, multiple intervention design for matched samples with the outcome defined to be ED visits where cannabis was listed as the primary diagnosis code**

|  | Depressed (A) | Non-depressed (B) | Difference test (A)-(B) |
| --- | --- | --- | --- |

|  | | | |
| --- | --- | --- | --- |
| **Female, matched sample** | | | |
| Pre-legalization trend | 0.016 (0.009 to 0.022) | 0.013 (0.003 to 0.022) | 0.003 (-0.008 to 0.014) |
| Phase 1 intercept change | 0.141 (-0.079 to 0.361) | 0.241 (-0.118 to 0.600) | -0.100 (-0.521 to 0.321) |
| Phase 1 slope change | -0.0149 (-0.036 to 0.006) | -0.012 (-0.044 to 0.019) | -0.002 (-0.040 to 0.035) |
| Phase 2 intercept change | 0.359 (0.041 to 0.677) | 0.483 (0.049 to 0.917) | -0.124 (-0.062 to 0.414) |
| Phase 2 slope change | -0.038 (-0.059 to -0.017) | -0.030 (-0.065 to 0.006) | -0.008 (-0.049 to 0.033) |
| **Male, matched sample** | | | |
| Pre-legalization trend | 0.017 (0.013 to 0.021) | 0.013 (0.003 to 0.022) | 0.005 (-0.006 to 0.015) |
| Phase 1 intercept change | -0.118 (-0.251 to 0.015) | -0.263 (-0.611 to 0.085) | 0.145 (-0.227 to 0.517) |
| Phase 1 slope change | -0.017 (-0.030 to -0.0053) | -0.012 (-0.034 to -0.01) | -0.005 (-0.031 to 0.020) |
| Phase 2 intercept change | 0.167 (-0.008 to 0.342) | 0.372 (-0.051 to 0.796) | -0.206 (-0.664 to 0.252) |
| Phase 2 slope change | -0.010 (-0.027 to 0.006) | -0.015 (-0.061 to -0.032) | 0.004 (-0.045 to 0.054) |
